# Traumatic Abdominal Wall Hernia With Sigmoid Colon Transection Following Bicycle Handlebar Injury: A Rare Case Report

**DOI:** 10.7759/cureus.107938

**Published:** 2026-04-29

**Authors:** Chaitanya P Garg, Wedad M Alsaumahi, Mohammed Q Aldulaimi, Muna O Alayyan, Qahtan A Aldulaimi

**Affiliations:** 1 Surgery, Saqr Hospital, Emirates Health Services, Ras Al Khaimah, ARE; 2 Surgery, RAK Medical and Health Sciences University, Ras Al Khaimah, ARE; 3 Faculty of Medicine, Alexandria University, Alexandria, EGY

**Keywords:** blunt abdominal trauma, computed tomography, hartmann’s procedure, hernia, laparotomy, sigmoid colon injury

## Abstract

Traumatic abdominal wall hernia may occur when a focal blunt force from a bicycle handlebar transmits substantial energy to the abdominal wall and viscera despite minimal external injury. This injury creates a diagnostic challenge, as early clinical findings can be subtle while serious intra‑abdominal injury evolves. We report a case of a 45‑year‑old man who presented with delayed, progressive abdominal pain after low‑velocity handlebar trauma. An early contrast‑enhanced CT was decisive in identifying a large abdominal wall defect with bowel herniation, free air, and suspected colonic perforation, prompting urgent surgical intervention. Exploratory laparotomy revealed near‑complete sigmoid colon transection with fecal contamination, requiring Hartmann’s procedure and abdominal wall repair. This case underscores that handlebar‑related abdominal trauma should lower the threshold for cross‑sectional imaging and early operative management, as timely intervention is critical to prevent missed hollow‑viscus injury and associated morbidity.

## Introduction

Blunt abdominal trauma is a major contributor to trauma-related health issues globally. While severe intra-abdominal injuries are typically linked to high-energy incidents such as motor vehicle collisions and falls, even low-velocity impacts can cause significant damage. A notable mechanism of injury is the impact from bicycle handlebars, which concentrates force over a limited area, thereby transmitting energy to deeper abdominal structures and raising the likelihood of visceral and abdominal wall injuries [1,2].

One rare yet clinically significant outcome of this mechanism is traumatic abdominal wall hernia (TAWH), characterized by a disruption in the abdominal wall muscles and fascia without any breach of the skin [1]. Bowel injuries occur in approximately 1% of patients with blunt abdominal trauma, with colonic involvement being relatively rare compared to small bowel injury [3]. Adult TAWH with concomitant colonic transection following low-velocity trauma, being extremely rare, remains sparsely characterized.

Due to potentially subtle external signs and patients often presenting with stable vital signs, the diagnosis might be delayed. Although focused assessment with sonography for trauma (FAST) can identify free intraperitoneal fluid, contrast-enhanced computed tomography (CT) is preferred for detecting defects in the abdominal wall, associated bowel injuries, and pneumoperitoneum [4]. Management becomes particularly complex when TAWH occurs alongside bowel injury and peritonitis, often necessitating bowel resection and stoma creation [1]. We report an unusual case of a TAWH due to bicycle handlebar injury associated with near-total transection of the proximal sigmoid colon in an adult male.

## Case presentation

A 45-year-old male was brought to the emergency department (ED) 12 hours after a bicycle accident. The patient mentioned that he had to use the brakes suddenly to avoid colliding with a car in front of him. This action caused him to fall over the handlebars of his bicycle and sustain injury on the left side of his abdomen. He had no injuries to the head or chest. He took analgesics from a nearby clinic and went home, but his abdominal pain progressively increased and spread all over the abdomen.

**Figure 1:**
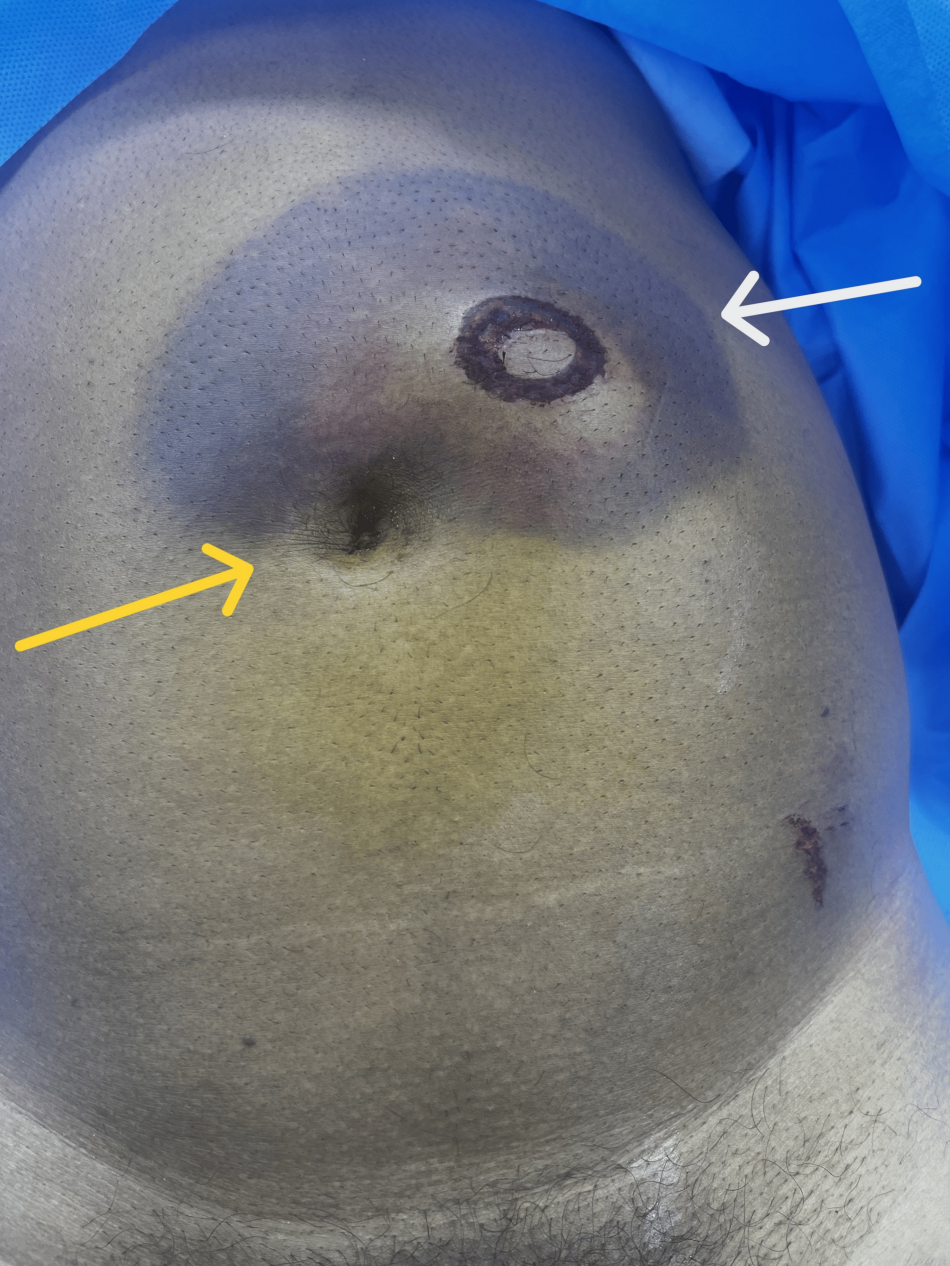
Preoperative image of the patient The image shows a handlebar injury mark with surrounding hematoma (white arrow) located above and to the left of the umbilicus (yellow arrow).

Upon arrival at the ED, he was fully alert and oriented. His vital signs were stable. On physical examination, his abdomen was distended with tenderness and guarding over the left lumbar region. There was a handlebar mark over the left side of the abdomen: to the left and above the umbilicus with a bruise and hematoma around it (Figure 1).

The rest of the abdominal regions were soft with normal bowel sounds. The patient’s body mass index (BMI) was 23 kg/m^2^, and he was a known diabetic on oral medications. Intravenous fluids and analgesics were given. In the laboratory investigations, the white blood cell count was 7.96 x 10^3^/mcL (range: 4.0-10.0), C-reactive protein was 42.6 mg/L (range: 0-3.0), blood glucose was 7.6 mmol/L (range: 3.9-7.8), and renal function tests were in the normal range.

**Figure 2:**
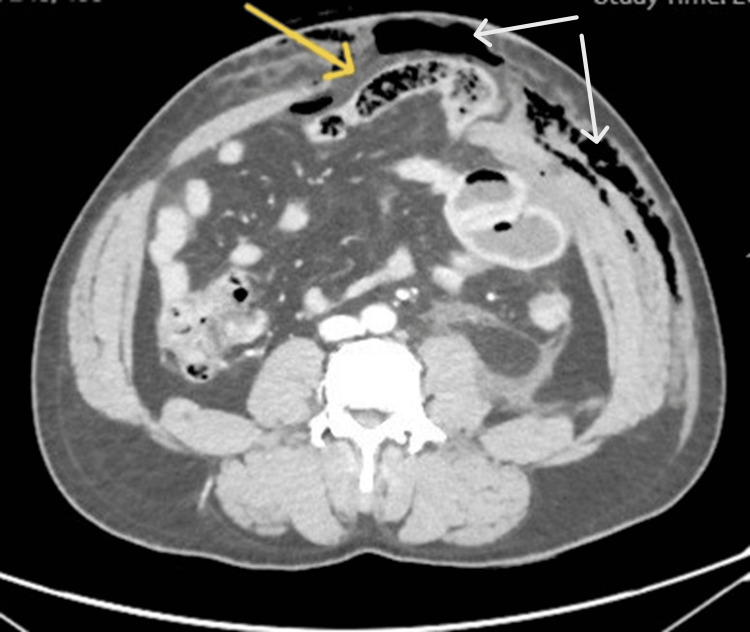
Computed tomography (CT) scan The image shows an abdominal wall defect with a herniating bowel loop (yellow arrow) and subcutaneous emphysema (white arrows), suggestive of bowel perforation.

Abdominal ultrasound demonstrated a small amount of fluid in the pelvis and normal solid organs. Contrast-enhanced CT scan of the abdomen and pelvis revealed a large left anterior abdominal wall defect involving the rectus abdominis muscle, measuring approximately 7 cm, with herniation of small bowel loops into the subcutaneous tissue and associated emphysema (Figure 2).

**Figure 3:**
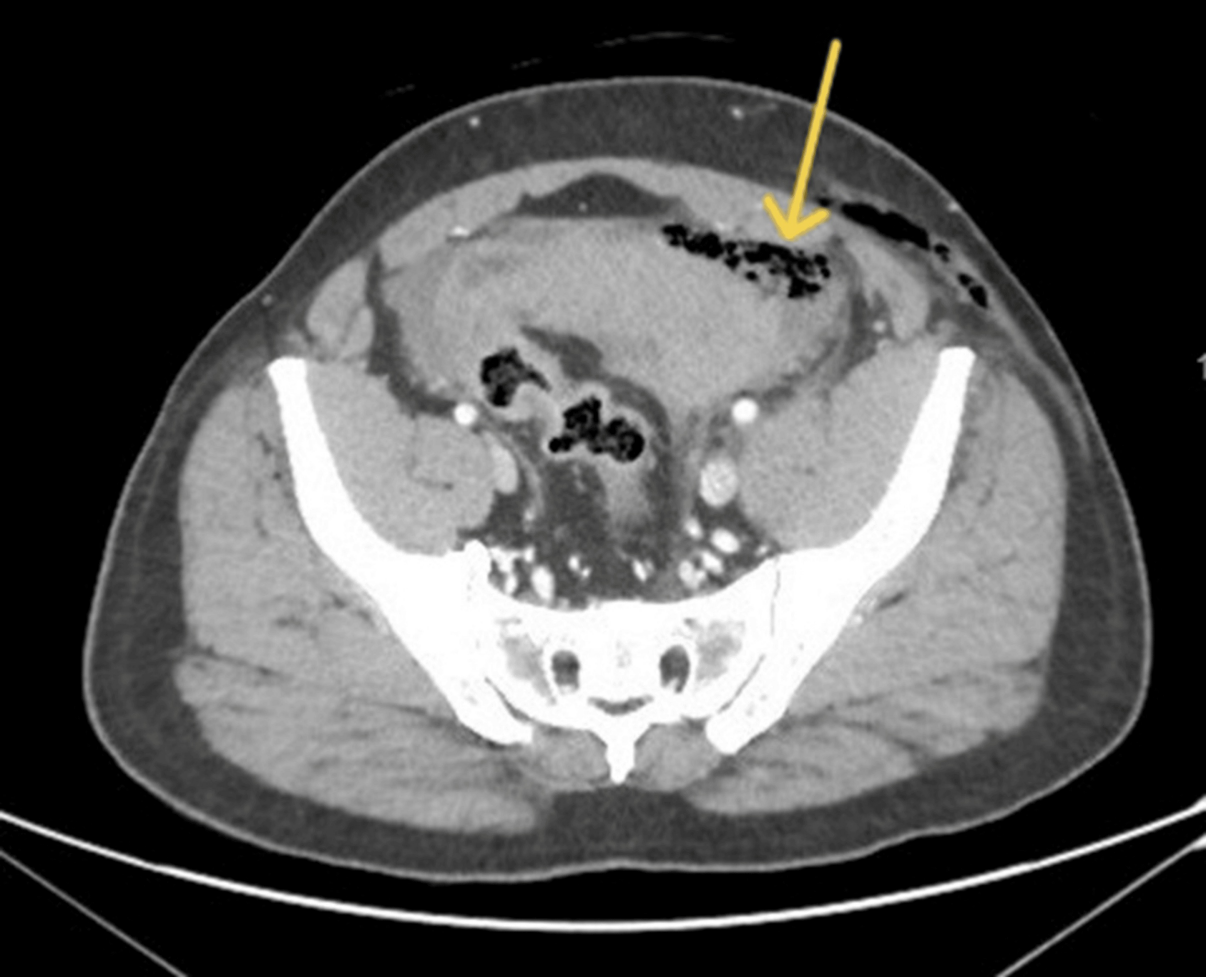
Computed tomography (CT) scan The image shows fecal contamination outside the bowel loops in the left lower abdomen (arrow), suggestive of colonic injury.

Mild intraperitoneal free fluid, free intraperitoneal air, and a 6 × 6 cm pelvic hematoma were also seen. The sigmoid colon showed features suspicious for fecal contamination, raising concern for bowel perforation (Figure 3).

**Figure 4:**
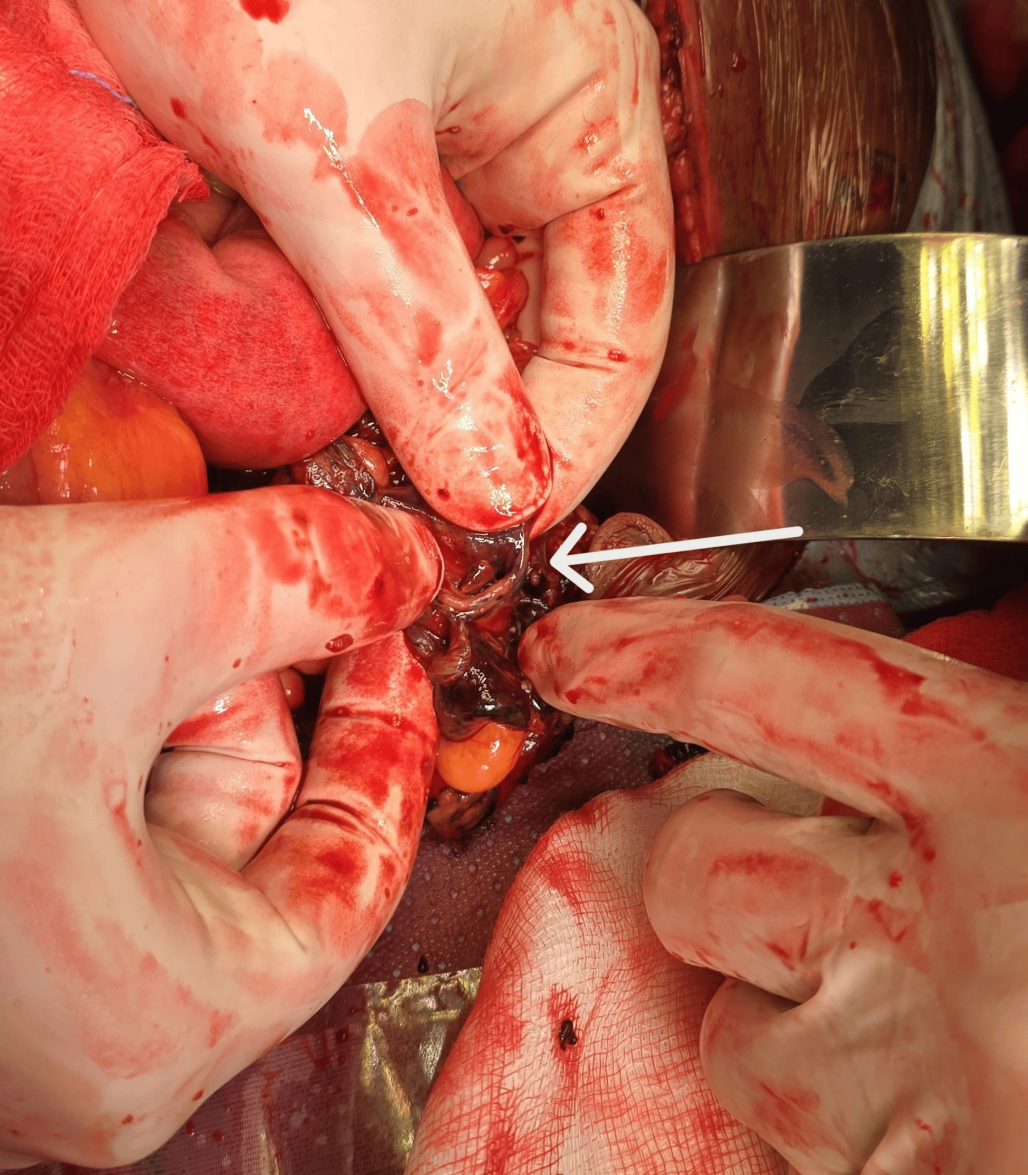
Intraoperative image The image shows a transected sigmoid colon (arrow).

Given the presence of abdominal distension, signs of peritonitis, and the patient’s delayed presentation, the decision was made to proceed with an emergency exploratory laparotomy. Intraoperatively, significant hemoperitoneum with large blood clots and fecal contamination, predominantly in the left iliac fossa, was found. A near-total transaction of the proximal sigmoid colon was identified (Figure 4).

There was minor oozing from a torn sigmoid mesentery artery, which was controlled using suture ligation. All intraperitoneal blood was evacuated, and a systematic assessment of the small bowel and remaining colon revealed no additional perforations. The stomach, liver, and spleen had no injuries.

**Figure 5:**
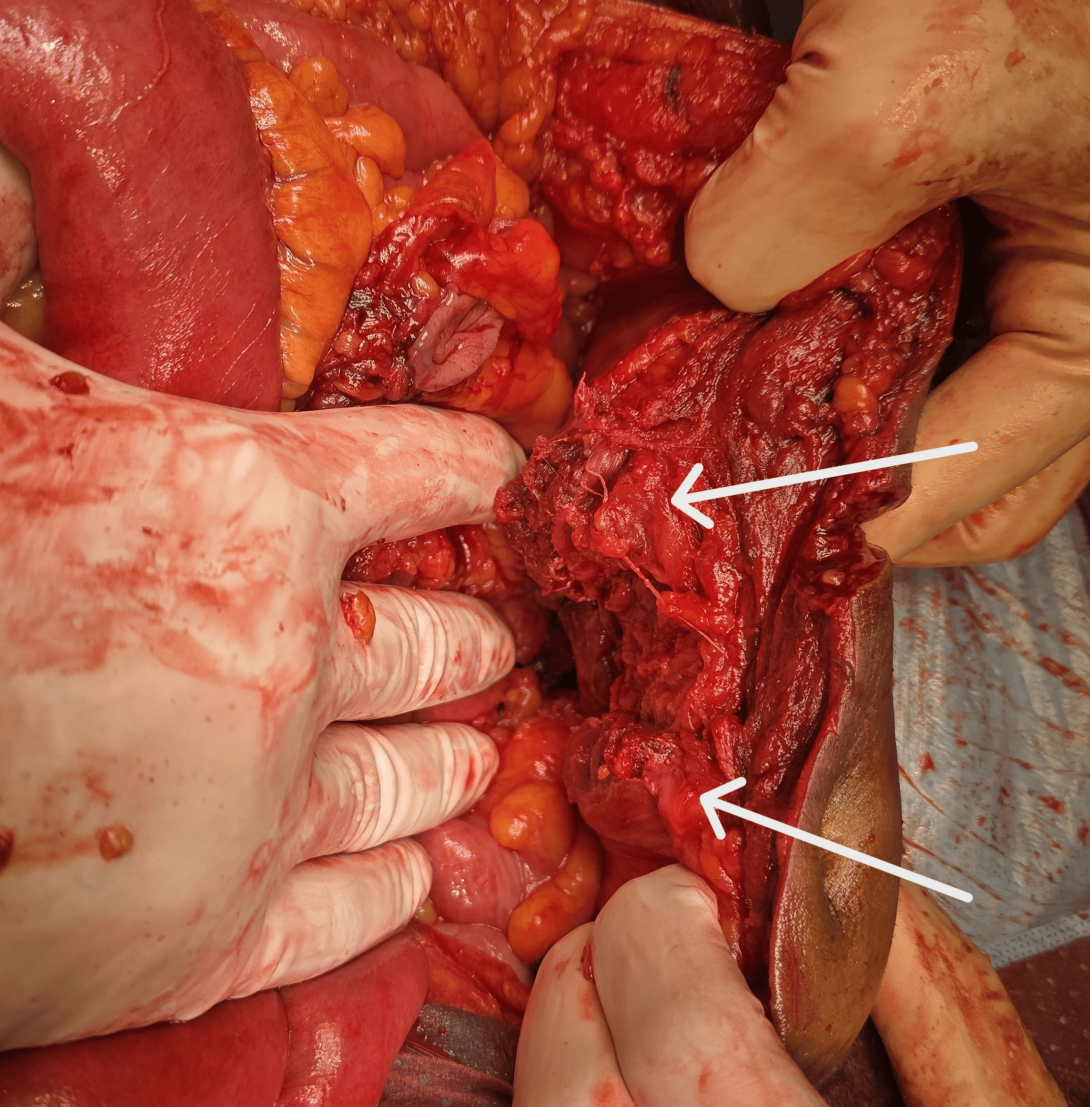
Intraoperative image The image shows a torn left rectus muscle (arrows).

The presence of fecal contamination, combined with tissue injury and mesenteric vascular disruption - even though bleeding was controlled - posed a high risk for anastomotic failure. Hence, a Hartmann’s procedure was preferred. The distal sigmoid colon was closed using a linear stapler and secured to the lateral abdominal wall. The proximal segment was mobilized and exteriorized as an end colostomy in the left upper abdomen. A transverse incision over the handlebar injury exposed a torn rectus muscle (Figure 5).

**Figure 6:**
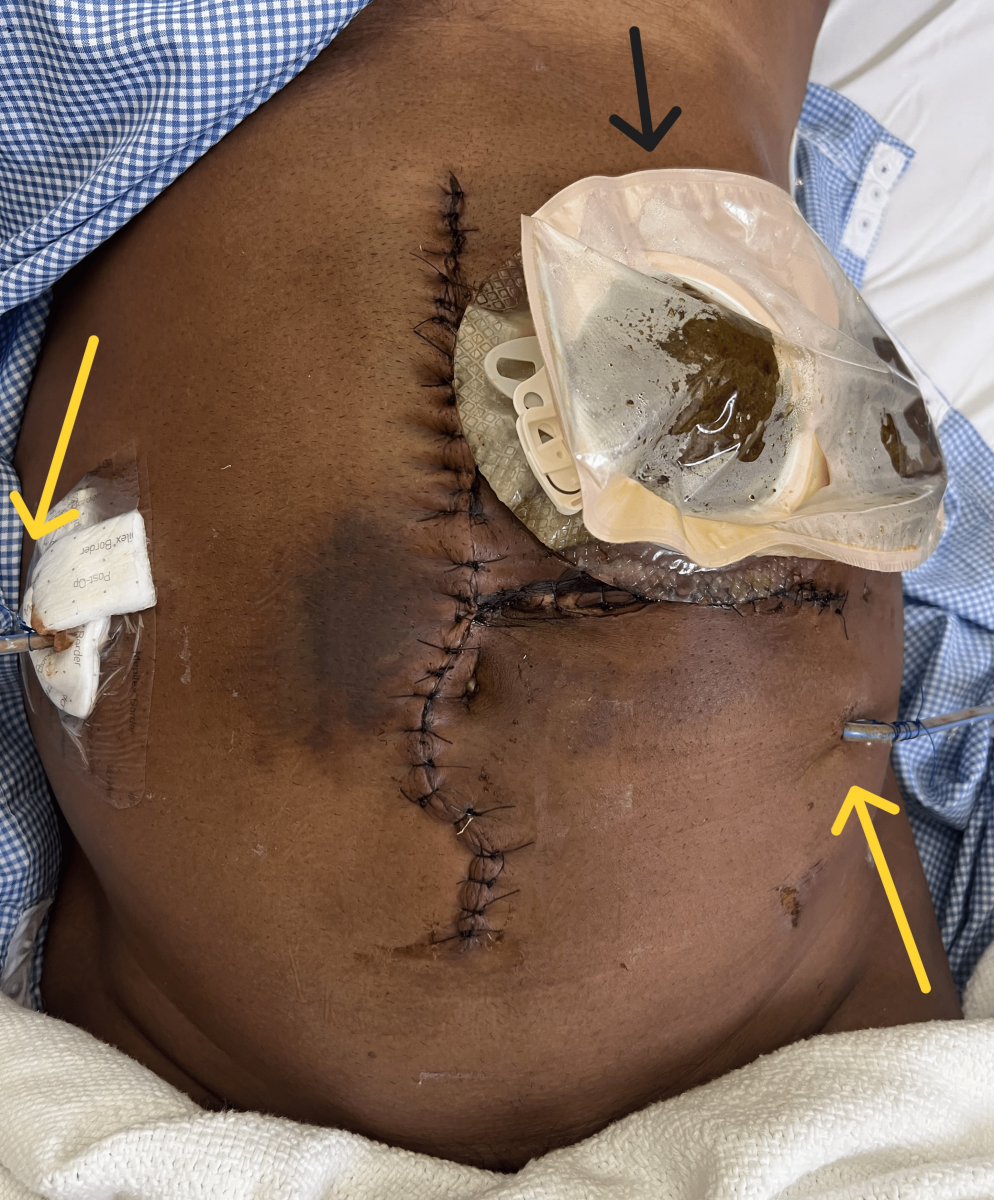
Postoperative image The image shows two intra-abdominal drains (yellow arrows) and a colostomy bag (black arrow).

The muscle was repaired in two layers using nylon in a continuous fashion. Thorough saline lavage was performed. Two intra-abdominal drains were placed, one in the pelvis and the other in the left iliac fossa (Figure 6).

**Figure 7:**
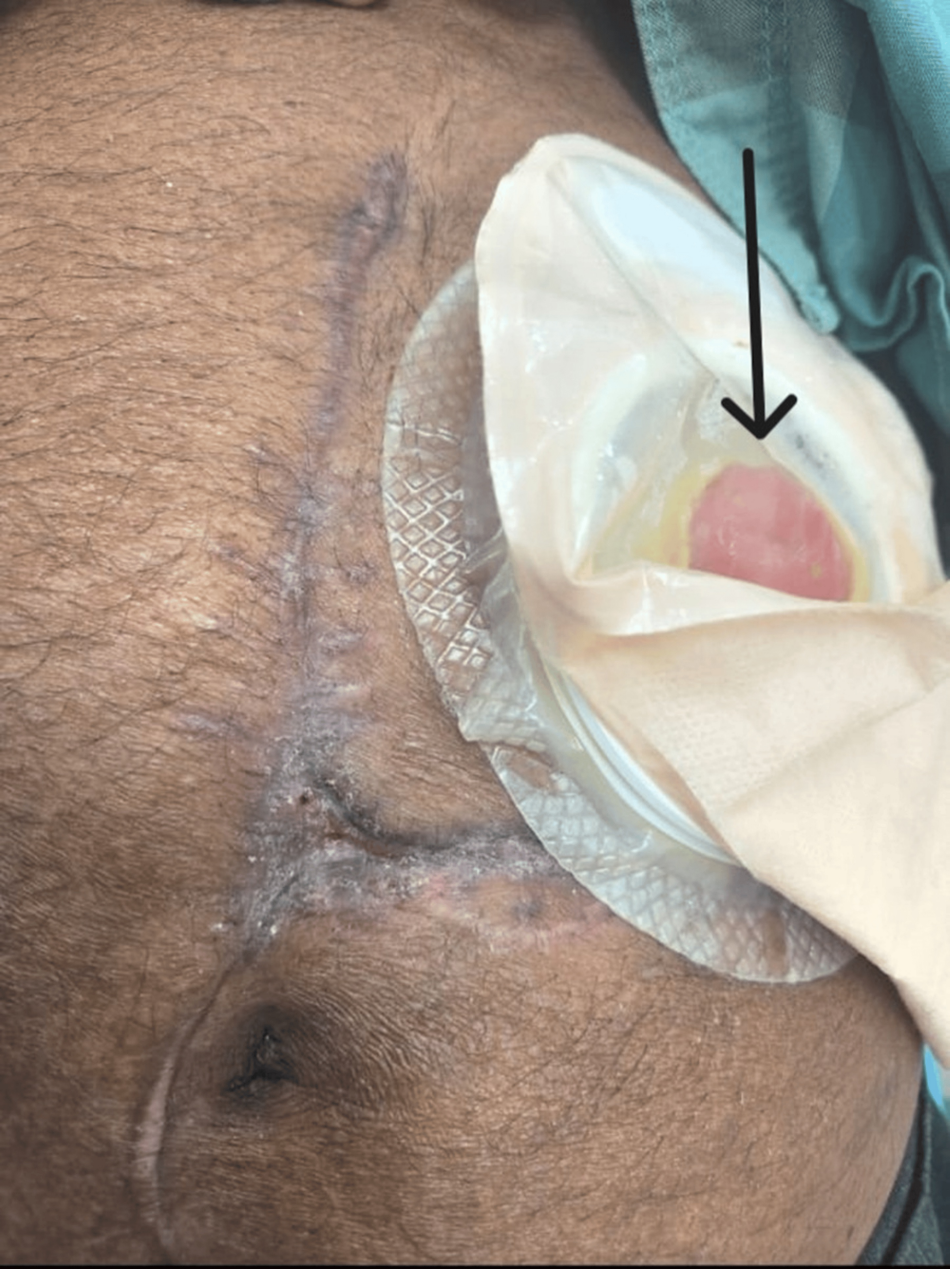
Postoperative patient image (after six months) The image shows a healed wound and colostomy (arrow).

Postoperatively, he had a good stoma function but developed superficial wound infection at the initial trauma site. He was discharged 12 days post-surgery with complete wound healing during follow-up in the outpatient clinic at six weeks (Figure 7). He is scheduled for colostomy closure after three months.

## Discussion

Bicycle handlebar injuries constitute a distinct form of blunt abdominal trauma in which low-velocity impact can result in disproportionate internal injury [4]. The narrow contact surface of the handlebar concentrates force over a small area, leading to a sudden rise in intra-abdominal pressure. This mechanism disrupts the underlying abdominal wall musculature and fascia while preserving skin integrity, resulting in TAWH. The force transmission to deeper structures frequently causes associated intra-abdominal injuries. Bowel perforation likely results from direct compression against the vertebral column combined with shear forces at fixed segments of the bowel [5]. Most cases occur in children due to thinner and less developed abdominal musculature [2]. Adult TAWH remains uncommon and is primarily described in isolated case reports and small case series, underscoring the rarity of this injury pattern in adults [6,7].

In a case series and accompanying literature review, Hefny et al. reported 10 adult cases of bicycle handlebar-related abdominal wall hernias. Among these, four patients sustained associated small‑bowel injuries and one had gastric injury; notably, no colonic injuries were identified [8]. More recently, an isolated case of ascending colon perforation following bicycle handlebar trauma was described in an 11‑year‑old boy, who presented two days after the injury and exhibited no associated abdominal wall hernia [9].

Patients often present with stable vital signs and minimal initial findings. Hemodynamic stability does not exclude significant intra-abdominal injury, as bowel injury may evolve with delayed peritonitis [3]. This deceptive presentation increases the risk of misdiagnosis or underestimation as a simple abdominal wall contusion or hematoma [1]. Accordingly, imaging plays a critical role in early diagnosis. Contrast-enhanced CT remains the modality of choice for suspected TAWH, as it accurately identifies abdominal wall defects, bowel injury, and pneumoperitoneum [1,2]. Prompt availability of CT imaging in our institution enabled comprehensive injury assessment and expedited surgical decision-making.

In cases involving colonic injury with fecal contamination, primary repair or anastomosis carries a high risk of anastomotic failure and leak-related morbidity. The World Society of Emergency Surgery (WSES) recommends end stomas and Hartmann’s procedure in such cases [10]. In our patient, Hartmann’s procedure provided effective source control and resulted in an uncomplicated recovery.

## Conclusions

Although bicycle handlebar trauma is often perceived as a low-energy mechanism, it can result in significant intra-abdominal injury. Early clinical assessment may be misleading, as patients frequently present with minimal symptoms and preserved hemodynamic stability. The presence of localized abdominal wall bruising following handlebar impact should therefore prompt a high index of suspicion and an early evaluation with contrast-enhanced CT. Surgical intervention is warranted when imaging or clinical findings indicate bowel injury or intra-abdominal contamination. In cases of colonic perforation associated with fecal contamination, Hartmann’s procedure remains a safe and effective operative strategy.
